# Prevalence of Depression, Suicidality, Alcohol Use Disorder and Associated Factors Among Adults on Antiretroviral Therapy in Namibia

**DOI:** 10.1007/s10461-025-04855-z

**Published:** 2025-09-03

**Authors:** Ndeshiteelela K. Conteh, Ozayr Mahomed

**Affiliations:** University of KwaZulu-Natal, Durban, South Africa

**Keywords:** HIV, Depression, Suicidality, Alcohol use disorder, Namibia

## Abstract

The increased risk associated with HIV infection and suicidality, depression, and alcohol use disorder underscores the need to treat mental illness in people living with HIV by integrating mental health services into routine HIV care. This study, conducted at nine health facilities providing antiretroviral treatment in Namibia from August and September 2022, aimed to determine the prevalence and factors associated with depression, suicidality, and alcohol use disorder among adults living with HIV/AIDS in Namibia. Amongst the 400 participants, the prevalence of depression, suicidality, and alcohol use disorder was 8%, 21%, and 24%, respectively. The prevalence of depression and suicidality was highest in females (9.7%) and (24.7%), respectively, while males had a prevalence of alcohol use disorder of 35.3%. Patients with depression showed an increased and significant risk of suicidality (aOR=8.280, 95% CI: 3.644–18.815, *p*=0.000). For alcohol use disorder, male patients (aOR= 2.995, 95% CI: 1.816–4.938, p=<0.000) were more likely to have alcohol use disorder. Male sex (aOR= 0.447, 95% CI: 0.240–0.832, p=<0.011), depression (aOR= 8.283, 95% CI: 3.644–18.828, p=<0.000), and alcohol abuse (aOR= 2.393, 95% CI: 1.337–4.285, *p*=0.003) had an increased and significant association with suicidality. Overall, the study’s results show that alcohol use disorder and suicidality are more prevalent in PLHIV compared to depression, and gender is a significant risk factor. Screening PLHIV and early initiation of treatment or interventions for various mental health disorders are important to improve retention, viral suppression, and other outcomes of ART.

**Supplementary Information:**

The online version contains supplementary material available at 10.1007/s10461-025-04855-z.

## Introduction

The emergence and increasing use of antiretroviral therapy (ART) has significantly altered the trajectory of HIV, shifting it from a previously fatal illness to a chronic condition that can be more effectively managed. This transformation has resulted in a decline in HIV-related mortality rates and an improvement in life expectancy, particularly in several countries within the sub-Saharan African region [1]. Due to a rise in HIV diagnoses, a growing number of individuals on antiretroviral therapy (ART), and the achievement of viral suppression, HIV programs have prioritized the enhancement of the quality of life for people living with HIV (PLHIV). The attainment of an improved quality of life for PLHIV necessitates the acknowledgment and management of the serious mental [2] health and substance abuse issues are prevalent among those who are living with HIV and those who are susceptible to contracting HIV. Evidence from many regions throughout the world also suggests that individuals living with HIV (PLWH) experience a higher prevalence of mental health issues when compared to the general population. A multisite study conducted in the United States involving a sample of 2864 adults receiving HIV care showed that about half of the participants exhibited positive screening results for depression. Additionally, nearly 40% of the participants reported engaging in illicit drug use, excluding marijuana, while approximately 12% screened positive for drug dependence within the preceding 12-month period [3]. whilst a national drug use and health survey conducted in the United States in 2003- 2004 found a prevalence of depression and drug use at 6.7% and 2.1% respectively in the general population [4]. A study conducted in PLHIV in Delhi, India, reports that 59% of PLHIV exhibited symptoms indicative of serious depression [5]. Findings from a systematic review study in China showed that 61% of people living with HIV (PLWH) exhibited signs of depression [6]. A study in the Entebbe district of Uganda showed that 14% out of 1099 ART-naïve PLWH (those living with HIV) were diagnosed with serious depression [7]. whilst in South Africa, it has been estimated that approximately 26-38% of People Living with HIV (PLWH) experience a mental problem, whereas the prevalence of mental disorders in the overall population is approximately 13% [8].

Namibia has an adult HIV prevalence of 12.6% [9], and HIV incidences and HIV-related deaths are reported to be reducing as access to ART increases and viral load suppression is attained [1]. Namibia has made great strides towards attaining the UNAIDS 90-90-90 goals as the 2017 NAMPHIA study findings have reported that in 2017, 86% of PLHIV are tested and know their status, among those that are tested and know their status, 96.4% are on ART and among those that are on ART, 93.1% are virally suppressed [9]. HIV prevalence varies across regions in Namibia, with the Zambezi region having the highest HIV prevalence of 22.3% reported and the lowest prevalence in the Kunene region (7.6%) [9]. Even with great strides made towards HIV epidemic control in Namibia, the country still has one of the highest prevalences of HIV, and the sub-Saharan region disproportionately bears the global burden of HIV [1]. Currently, there is limited information on the prevalence of mental health illnesses amongst PLHIV in Namibia, as the services are not integrated. Assessing the prevalence of depression, alcohol use disorder, and suicidality and identifying factors associated with them will yield valuable insights for developing targeted clinical interventions.

The purpose of this study was to determine the prevalence of depression, suicidality, and alcohol use disorder in adult PLHIV as well as factors associated with depression, suicidality, and alcohol use disorder in that population.

## Methods

### Study Design and Population

The study was an analytical cross-sectional study that was conducted in Windhoek, the capital city of Namibia Windhoek All PLHIV 18 years and older in Windhoek, Khomas region, who were active on ART between August and October, were eligible for inclusion in the study. There were 20,173 patients active on ART in the database. A representative sample of adults aged 18 years receiving ART in Windhoek, covering the period from August to October 2022. Using the national electronic patient monitoring system (ePMS), we identified 20,173 patients active on ART in Windhoek during this period. Based on previous research indicating a 30% prevalence of mental illness among People Living with HIV (PLHIV) in sub-Saharan Africa [17], and using a correlation factor of two, the minimum required sample size needed to reliably estimate the prevalence of mental illness with a 95% confidence level, 80% power, and a 5% margin of error were 318 individuals. For increased precision, we rounded the target sample size to 400 and proportionately allocated it across the nine health facilities. A total of 9 public health facilities were included in the study, and 2 were excluded from the study. The two health facilities excluded from the study had a combined total of 4 patients actively receiving ART care and are located outside the city; therefore, they were excluded from the study for logistical considerations. After the sample was proportionally allocated across sites, we used the Rand Between function in MS Excel to randomly select eligible patients from the active patient list in the ePMS for each site. In Namibia, a patient “active on care” typically refers to a patient who is actively participating in and benefiting from the ART program and is not lost to follow-up.

### Study Sample

A two-stage stratified random sampling design was applied to select participants. First, all 12 public health facilities providing ART services were included using probability proportional to size (PPS) sampling, based on the number of patients actively on ART at each site. The overall target sample size was 400 patients, calculated using a single population proportion formula. In the second stage, the total sample was proportionally allocated across the selected health facilities. From each facility’s active patient list in the Electronic Patient Monitoring System (ePMS), eligible patients were randomly selected using the Rand function in Microsoft Excel. The sampling frame was filtered to include patients who had ART visits during the study period. Finally, a sampling list of selected active patients by health facility was prepared. At each facility, this list was shared with healthcare workers, who then referred the identified patients to participate in the study. Patients were identified using their unique ART code.

### Data Collection

The mini-international neuropsychiatric test (MINI) tool is a concise and organized diagnostic interview instrument that adheres to worldwide diagnostic standards, such as the International Classification (ICD-10), Diagnostic and Statistical Manual of Mental Disorders, Third Edition Revised (DSM-III-R), and Fourth Edition (DSM-IV) [10] was utilized. The licensing organization trained the researcher on how to administer the tool. The Mini-International Neuropsychiatric Interview (M.I.N.I.) is a brief, structured diagnostic tool used to assess psychiatric disorders based on DSM-IV and DSM-5 criteria. It uses yes/no symptom screening and a scoring system to identify conditions such as depression, alcohol use disorder (AUD), and suicidality. Depression is classified based on core symptoms like low mood and loss of interest, AUD through patterns of harmful alcohol use, and suicidality by assessing thoughts, plans, or attempts of self-harm. The M.I.N.I. is quick to administer—typically in 15 min—yet provides reliable and valid results, making it ideal for both research and clinical settings [11].

Demographic data were collected using a patient demographic questionnaire that the research assistant administered. We collected data on age, sex, marital status, education level, employment status, sexual orientation, and duration of ART. The researcher recruited 4 research assistants with at least 2 years of experience working in a health setting as data collectors. The research assistants were required to speak one or all the main spoken languages (English, Oshiwambo, and Afrikaans. All participant interviews were done at the health facilities.

### Statistical Analysis

Data management was conducted in Microsoft Excel, and statistical analyses were performed using STATA version 15.1 (Stata Corp, 2017). The three primary outcome variables were: (1) depression, (2) alcohol use disorder, and (3) suicidality. Independent variables were sex, age group, marital status, gay/lesbian sexual orientation, and the period the patient had been on ART. These independent variables were selected based on associations identified in previous studies ADDIN EN.CITE [5, 7, 12–15] their availability in the ePMS, and their perceived utility/practicality for future behavioral interventions. sex, age group, marital status, gay/lesbian sexual orientation, and the period the patient has been on ART. We used the Chi-square to test the associations between each independent variable and the outcome variable. Variables that were p < 0.05 level of significance were entered into bivariable logistic regression models, with separate models for each of the three outcomes. In each of the three models, we included the other two primary outcome variables (if significant) as possible predictors. For example, in one model we assessed whether, when controlling for other variables, alcohol or suicidality predicted depression, in the second model we assessed whether depression or suicidality predicted alcohol use, and in the third model we assessed whether alcohol use or depression predicted suicidality. Variables with p-values p *<* 0.1 in the bivariable logistic regression models were entered into multivariable models. Variables with p-values *<* 0.05 in multivariable models were considered statistically significant.

### Ethical Considerations

Ethical Approval for the study was granted by the University of KwaZulu-Natal’s Biomedical Research and Ethics Committee (ref: BREC/00002904/2021) as well as the Ministry of Health and Social Services’ ethical committee (Ref: 17/3/NKC). Study respondents gave written consent using pseudonyms to participate in the study. Participants were given the choice to withdraw from the study at any point if they wished to, as well as skip any questions that they were not comfortable answering. No personally identifying information, like first and last names or identity numbers, was collected to ensure the anonymity of participants. All individuals who screened positive for suicidality, depression, or alcohol use disorder were directed to a healthcare provider for further evaluation or referred for appropriate care per the Ministry of Health and Social Services’ standard procedures.

## Results

### Socio-demographic Characteristics of Participants

The study enrolled its target sample of 400 patients attending ART care in nine public health facilities in Windhoek. Most of the patients (66.8%) were females, and 33.3% were males. Of the 400 participants. Almost 75% of participants reported that they have received secondary education, whilst 53.3% of the participants were unemployed. The median age of participants was 38 years old (SD 10.74 The minimum age of participants was 18 years, and the highest age was 66 years. Most participants were from the age group 40-44 years old (17.8%) in the age groups 35-39 (60/400) and 45-49 (63/400) (Table 1). Among all 400 participants on ART, 69.3% were on ART for 5 years or more, 25.7% were on ART for 1-5 years, 3.0% were on ART for 4-12 months, and 2.0% were on ART for less than 3 months (Table 1). Only 2% of participants were newly initiated in 3 months or less (Table 1). Among all participants, 76.3% reported that they were single, 22.5% were married, 0.5% were divorced, 0.3% were separated, and 0.5% were widowed. Only 1% of participants identified as gay or lesbian (Table 1).

**Table 1:** Socio-demographic characteristics of participants

Variable	*N* (%)
**Age group**	
15-19	21 (5.25)
20-24	36 (9%)
25-29	42 (10.5)
30-34	52 (13%)
40-44	71 (17.75%)
45-49	63 (15.75%)
50-54	33 (8.25%)
55-59	16 (4%)
60+	6 (1.5%)
**Sex**	
Female	267 (66.75%)
Male	133 (33.35%)
**Marital Status**	
Divorced	2 (0.5%)
Married	90 (22.5%)
Separated	1 (0.25%)
Single	305 (76.25%)
Widowed	2 (0.5%)
**Education Attainment**	
No education	18 (4.56%)
Primary Education	43 (10.89%)
Secondary Education	295 (74.68%)
Tertiary Education	39 (9.87%)
**Employment**	
No	212 (53.27%)
Yes	186 (46.73%)
**Income (NAD)**	
<1000	6 (1.50%)
1000-2000	32 (8%)
2000-4,500	87 (21.75%)
4,5000-10,000	38 (9.50%)
10,000-20,000	9 (2.25%)
>20,000	14 (3.50%)
Missing	214 (53.5%)
**Gay/Lesbian sexual identity**	
No	393 (98.99%)
Yes	4 (1.01%)
**Duration on ART**	
<3 months	8 (2.02%)
4-12 months	12 (3.02%)
1-5 years	102 (25.69%)
>5 years	275 (69.27%)

### Prevalence and Factors Associated with Depression

Thirty-one participants out of 400 (8%) screened positive for depression. The prevalence of depression was higher in females than in males, where 9.7% of females (26/267) had depression compared to 3.8% of males (5/133). By age, the prevalence of depression was similar across all three age groups, although the age group 25-49 reported a prevalence of almost 8% (23/288). Depression was also relatively higher among unmarried participants who reported a prevalence of 8.7% (27/310), 8.2% of those who had primary education or lower (5/39), and 9.4% of those who were unemployed (20/212). (Table 2). Male sex was significant at a 5% level of significance in bivariate analysis. Also, patients with suicide ideation (cOR: 10.200, 95% CI: 4.581-22.709, P-value: 0.000) and alcohol use disorder (cOR: 2.489, 95% CI: 1.171-5.290, P-value: 0.018) are at an increased risk of having depression. However, after adjusting for possible confounders (age, marital status, sexual orientation, education, employment, income, duration on ART), the odds of depressed patients on ART having suicidal ideation were 8.2 times higher when compared to patients who were not depressed. Other factors such as sex, age group, marital status, educational attainment, income, and duration of ART were not associated with depression at a 5% level of significance when confounders were adjusted. (Table 3).

### Prevalence and Factors Associated with Suicidality

Eighty-four participants out of 400 (21%) screened positive for suicidality. The prevalence of suicidality was higher in females than in males, as 24.7% of females (66/264) screened positive for suicidality compared to 13.5% of males (18/133). The highest prevalence of suicidality was reported among participants aged 25-29 years, who had a prevalence of 22.92% (66/288) and among 21.6% % of participants who were unmarried (67/310) (Table 3). Additionally, participants with secondary education or higher reported the highest prevalence of suicidality of 22.5% (75/334) compared to 14.6% of participants with primary education or less (9/61) (Table 2). After adjusting for possible confounders (age, marital status, income, marital status, income and duration on ART) at multivariable analysis, only male sex (aOR= 0.477, 95% CI: 0.240-0.831, p=<0.011), being depressed (aOR= 8.283, 95% CI: 3.644-18.828, p=<0.001), and alcohol abuse (aOR= 2.393, 95% CI: 1.337-4.285, *p*=0.003) showed an increased association with suicide ideation (Table 3).

### Prevalence and Factors Associated with Alcohol Use Disorder

Ninety-six participants out of 400 (24%) screened positive for alcohol use disorders. Females had a lower prevalence of alcohol use disorder compared to males, where the prevalence among females was 18.35% (49/267) and males had a prevalence of 35.3% (47/133) (Table 2). The prevalence of alcohol abuse disorder was highest among the age group 25-49, where 25% (72/288) of participants screened positive for alcohol use disorder. Likewise, the highest prevalence of alcohol abuse disorder was reported among 25% of unmarried participants (80/310) and 24.3% of participants with secondary education or higher (81/334). Interestingly, the prevalence of alcohol use disorder was about the same among employed (24.2%) and unemployed participants (23.6%). (Table 2). Male sex (OR: 2.431, 95% CI: 1.517-3.897, p = <0.001) showed an increased odds or likelihood of having alcohol abuse at bivariate analysis. Having suicidal ideation (OR: 2.259, 95% CI: 1.342- 3.801, *p*=0.002) and depression (OR: 2.489, 95% CI: 1.171-5.290, *p*=0.018) were associated with increased odds of alcohol use disorder. After adjusting for possible confounders (age, sexual orientation, marital status, employment, income, duration on ART) at multivariable analysis, males compared to their female counterparts (OR: 2.995, 95% CI:1.816-4.938, p=<0.001), patients with suicidal ideation (OR: 2.394, 95% CI: 1.337-4.286, *p*=0.003) and depression (OR:2.105, 95% CI:0.912-4.859, *p*=0.08 were more likely to screen positive for alcohol use. Other factors such as age group, marital status, educational attainment, income, and duration on ART were not associated with alcohol abuse at 5% level of significance (Table 3).

Male sex (OR: 2.431, 95% CI: 1.517-3.897, p = <0.001) showed an increased odds or likelihood of having alcohol abuse at bivariate analysis. Having suicidal ideation (OR: 2.259, 95% CI: 1.342- 3.801, *p*=0.002) and depression (OR: 2.489, 95% CI: 1.171-5.290, *p*=0.018) were associated with increased odds of alcohol use disorder. After adjusting for possible confounders (age, sexual orientation, marital status, employment, income, duration on ART) at multivariable analysis, males compared to their female counterparts (OR: 2.995, 95% CI:1.816-4.938, p=<0.001), patients with suicidal ideation (OR: 2.394, 95% CI: 1.337-4.286, *p*=0.003) and depression (OR:2.105, 95% CI:0.912-4.859, *p*=0.08 were more likely to screen positive for alcohol use Other factors such as age group, marital status, education attainment, income, and duration on ART were not associated with alcohol abuse at 5% level of significance (Table 3).

## Prevalence of Depression, Suicidality, and Alcohol Use Disorder

See Tables 2 and 3.

**Table 2:** Prevalence of depression, suicide, and alcohol use disorder among adults receiving ART at public health facilities in windhoek, 2022

Variable	Depression, *n* (%)	Suicidality, *n* (%)	Alcohol use, *n* (%)
All participants (*N*=400)	31 (7.8%)	84 (21.0%)	96 (24.0%)
**Age group**			
18-24 (*n*=57)	4 (7.02%)	9(15.79%)	12 (21.05%)
25-49 (*n*=288)	23(7.99%)	66 (22.92%)	72 (25%)
50+ (*n*=55)	4 (7.27%)	9 (16.36%)	12 (21.82%)
**Sex**			
Female (*n*=267)	26 (9.74%)	66 (24.72%)	49 (18.35%)
Male (*n*=133)	5 (3.76%)	18 (13.53%)	47 (35.34%)
**Marital Status**			
Married (*n*=90)	4 (4.44%)	17 (18.89%)	16(17.78%)
Unmarried (*n*=310)	27 (8.71%)	67(21.61%)	80 (17.77%)
**Education Attainment**			
Primary Education or lower (*n*=61)	5 (8.2%	9 (14.75%)	5 (27.8%)
Secondary Education or higher (*n*=334)	26 7.78%)	75 (22.46%)	9 (25.81%)
**Employment**			
No (*n*=212)	20 (9.43%)	44 (20.75%)	50 (23.6%)
Yes (*n*=186)	10 (5.38%)	39 (20.97%)	45 (24.2%)
**Income (NAD)**			
<1000 (*n*=6)	0 (0%)	3 (50%)	2 (33.3%)
1000-2000 (*n*=32)	1 (3.12%)	5 (15.62%)	6 (18.75%)
2000-4,500 (*n*=87)	3 (3.45%)	17 (19.54%)	21 (24.14%)
4,500-10,000 (*n*=38)	3 (7.89%)	7 (18.42%)	9 (23.68%
10,000-20,000 (*n*=9)	0 (0%)	2 (22.22%)	3 (33.33%)
>20,000 (*n*=14)	3 (21.43%)	5 (35.71%)	4 (28.57%)
**Gay/Lesbian sexual identity**			
**No** (*n*=393)	31 (7.89%)	82 (20.87%)	91 (23.2%)
**Yes** (*n*=4)	0 (0%)	1 (25%)	4 (100%)
**Duration on ART**			
<1 year (*n*=20)	2 (10%)	4 (20%)	5 (25%)
1-5 years (*n*=102)	6 (5.88%)	16 (15.69%)	28 (27.45%)
>5 years (*n*=275)	23 (8.36%)	64 (23.27%)	62 (22.55%)

**Table 3:** Factors associated with depression, alcohol use disorder, and suicidality

	Total Patients	Number with disorder	Univariate		Multivariate	
Independent variables	400		cOR (95% CI)	*P* val.	aOR (95% CI)	*P* val.
For outcome: **Depression**		**32**				
Ref: Females		26				
Male		5	0.362 (0.136-0.966)	0.042*	0.403 (0.141-1.156)	0.091
Ref: 18-24		4				
25-49		23	1.150 (0.382-3.462)	0.804		
50+		4	1.039 (0.247-4.378)	0.958		
Ref: Unmarried		27				
Married		4	0.488 (0.166-1.432)	0.191		
Ref: Primary Education or Lower		5				
Secondary Education or Higher		26	0.945 (0.348-2.566)	0.912		
Ref: Employed No		20				
Yes		10	0.545 (0.249-1.197)	0.131		
Ref: Income <10 000		7				
10 000+		3	3.00 (0.722-12.466)	0.131		
Ref: ART <1 year		2				
1-5 years		6	0.563 (0.105-3.011)	0.501		
>5 years		23	0.821 (0.179-3.763)	0.800		
Ref: No Alcohol Use						
Alcohol Use Disorder			2.489 (1.171-5.290)	0.018*	2.107 (0.910)	0.082
Ref: No Suicidality						
Positive Suicidality			10.200 (4.581-22.709)	<0.001*	8.280 (3.644	<0.001*
For outcome: **Suicidality**		**84**				
Ref: Females		66				
Male		18	0.477 (0.270-0.42)	0.011*	0.447 (0.240)	0.011*
Ref: 18-24		9				
25-49		66	1.586 (0.739-3.401)	0.236		
50+		9	1.043 (0.381-2.861)	0.934		
Ref: Unmarried		67				
Married		17	0.845 (0.467-1.528)	0.577		
Ref: Primary Education or Lower		9				
Secondary Education or higher		75	1.673 (0.788-3.552)	0.180		
Ref: Employment No		44				
Yes		39	1.013 (0.624-1.645)	0.958		
Ref: Income <10 000		32				
10 000+		7	1.568 (0.603-4.074)	0.356		
Ref: ART <1 year		4				
1-5 years		16	0.744 (0.220-2.517)	0.635		
>5 years		64	1.213 (0.392-3.759)	0.738		
Ref: Not depressed			10.200 (4.581-22.079)	<0.001*	8.283 (3.644-18.828)	<0.001*
Ref: No Alcohol Use Disorder						
Alcohol Use Disorder			2.259 (1.342-3.801)	0.002*	2.393 (1.337-4.285)	0.003*
For outcome: **Alcohol Use Disorder**		**96**				
Ref: Females		49				
Male		47	2.431 (1.517-3.897)	<0.001*	2.995 (1.816-4.938)	<0.001*
Ref: 18-24		12				
25-49		72	1.250 (0.627-2.493)	0.526		
50+		12	1.047 (0.424-2.581)	0.921		
Ref: Unmarried		80				
Married		16	0.622 (0.342-1.130)	0.119		
Ref: Primary Education or Lower		14				
Secondary Education or higher		81	1.075 (0.563-20.53)	0.827		
Ref: Employment No		50				
Yes		45	1.034 (0.652-1.641)	0.887		
Ref: Income <10 000		37				
10 000+		8	1.577 (0.630-3.946)	0.330		
Ref: ART <1 year		5				
1-5 years		28	1.135 (0.377-3.416)	0.822		
>5 years		62	0.873 (0.305-2.498)	0.800		
Ref: Not depressed						
Depressed			2.489 (1.171-520)	0.018*	2.106 (0.913-4.859)	0.081
Ref: No Suicidality						
Positive Suicidality			2.259 (1.342-3.801)	0.002*	2.394 (1.337-4.286)	0.003*

****Significant: <0.005****

***cOR: Crude Odds Ratio

***aOR: Adjusted Odds Ratio

## Discussion

In a cross-sectional study conducted at nine public health facilities in Windhoek, Namibia, involving 400 adults living with HIV/AIDS who sought antiretroviral therapy (ART) services, the prevalence of mental health issues was notable. The findings indicated that 8% of participants experienced depression, 21% had suicidality, and 24% suffered from alcohol use disorder.

The prevalence of depression among PLHIV in this study is 8%, similar to the findings of a study conducted in Uganda and Zambia, which found the prevalence of depression to be at 8.1% and 7% respectively [7, 16] whilst other cross-sectional studies conducted in other contexts outside of Sub-Saharan Africa have found higher prevalences of depression among PLHIV at 28.2% [12]. Our study reported a higher prevalence of depression in females (9.74%) compared to males (3.76%), which is a similar trend reported by a cross-sectional study conducted at the Cape Coast Teaching Hospital in Ghana, which found that females had a higher prevalence of depression ADDIN EN.CITE [14]. Some studies have underscored that females have a higher risk of depression from youth to adulthood compared to males ADDIN EN.CITE [17–19]. Various external stressors and hormonal factors have been suggested to have a greater propensity to precipitate depression in women compared to men. Women not only exhibit a higher likelihood of reporting a significant life stressor in the six months preceding a major depressive episode but may also display increased susceptibility to depression following such stressors. Emerging evidence indicates that specific hormonal fluctuations related to reproductive processes contribute to elevating the risk of depression in females [20]. Consequently, depressive symptoms or major depressive episodes may manifest during premenstrual phases, postpartum periods, and premenopausal stages [18]. Also, the cross-sectional study in Ghana found female sex to have a statistically significant association with depression ADDIN EN.CITE [14]. While the cross-sectional study conducted in Ghana aligns with our findings regarding the significant association between gender and depression, it diverges from our results in one key aspect. In our study, the male sex was statistically significant for depression at the univariate level, whereas the Ghanaian study identified the female sex as significant. This inconsistency is echoed in the broader literature. A systematic and meta-review analysis from Sub-Saharan Africa indicates that there is no definitive consensus on whether male or female sex is conclusively associated with depression among people PLHIV [21], underscoring the complexity of gender dynamics in mental health outcomes within this population. Our study findings have not reported any significant association with any demographic variables, such as sex, marital status, employment status, and income; however, various studies have found an association with two demographic variables: poor economic status ADDIN EN.CITE [22, 23] and being less educated [21].

Interestingly, our study findings indicate that patients with alcohol use disorder are at a significantly increased risk of experiencing depression (cOR: 2.489, 95% CI: 1.171-5.290, P-value: 0.018). This aligns with findings from a cross-sectional study conducted in Chennai, India, among PLHIV, which similarly reported that current alcohol use was associated with more than twice the odds of developing a psychosocial condition ADDIN EN.CITE [24]. Additionally, research findings established a strong association between substance use and depressive symptoms [21] ADDIN EN.CITE [25, 26]. It is important to highlight that, in Sub-Saharan Africa, poor social conditions appear to be the predominant factor associated with depressive symptoms among PLHIV on antiretroviral therapy (ART) [21]. While demographic factors such as age, gender, and marital status show variability across different contexts, no definitive conclusions can be drawn due to the limited number of studies in this area. This underscores the complexity of understanding depression in PLHIV and highlights the need for more region-specific research to address these gaps.

This study’s findings report an overall prevalence of suicidality to be at 21%. The prevalence of suicidality is comparable to a pooled prevalence of 23.3%, which was reported in a systematic review that included various studies that assessed suicidality among PLHV in both low and high-income countries [27]. Similarly, another systematic review study that was conducted in low and high-income countries reports a pooled prevalence of suicidality among PLHIV of 20.4% [28]. The systematic review did not find any demographic factors to be significantly associated with suicidality among PLHIV [27]. These findings are consistent with our study, where male sex was the only demographic factor significantly associated with suicidality. All other demographic variables, both at the univariate and multivariate levels, were found to be insignificant. This suggests that, at least in our sample, male PLHIV may be at a heightened risk for suicidality, while other factors such as age, education, and marital status did not show a meaningful association.

Our study findings report that male sex (aOR= 0.447, 95% CI: 0.270-0.842, p=<0.011), being depressed (aOR= 8.283, 95% CI: 3.644-18.828, p=<0.000), and alcohol abuse (aOR= 2.393, 95% CI: 1.337-4.285, *p*=0.003) to be associated with suicide ideation. These findings are similar when compared to studies that assessed suicidality among PLHIV. For instance, the systematic review and meta-analysis by Tsai et al. identified substance abuse and depression as significant factors associated with suicidality among PLHIV, though other variables such as low quality of life and limited social support were also highlighted [27]. Depression has been consistently reported as a key factor linked to suicidality in studies examining its prevalence in PLHIV ADDIN EN.CITE [29–32]. In our study, being male was found to be associated with an increased risk of suicidality. Although the existing literature on why males are at heightened risk for suicidality remains limited, some studies suggest that traditional masculine norms, such as self-reliance and emotional stoicism, may place men at greater risk compared to women ADDIN EN.CITE [27, 28]. These gendered expectations may contribute to men’s reluctance to seek help, thereby increasing their vulnerability to mental health challenges.

The prevalence of alcohol use disorder in this study is 24%, which is higher compared to other studies conducted in both low- and middle-income country settings and high-income country settings. For example, a study by Rukundo et al. found the prevalence of alcohol use disorder in PLHIV in Uganda to be 15.1% [33] while a systematic and meta-analysis study reported that South Africa has an average prevalence of alcohol use disorder of 28.77% ADDIN EN.CITE [34]. Furthermore, the systematic study findings report that the prevalence of alcohol use varied with sample size. Studies with a sample size >450 had an average prevalence of alcohol use disorder of 16.71%, while studies with a sample size < 450 had an average prevalence of 26.46% [35] which makes this study’s findings comparable to studies with similar sample sizes that had a sample size of 400. Our study findings found male sex to have a significant association with alcohol use disorder. The results of this study are similar to the findings of a cross-sectional study conducted among PLHIV in Nairobi, Kenya, that found male sex to have a significant association with alcohol use disorder [36]. The odds of abusing alcohol were 2.995 for males compared to females. The association between being male and an increased risk for alcohol use disorder is supported by numerous studies that reported a similar association ADDIN EN.CITE [32, 37–40], which underscores that HIV-positive males are more likely to abuse alcohol than HIV-positive females. Gender differences in alcohol consumption are well-documented, and these differences are shaped by various cultural factors [41]. Studies have shown that male drinkers tend to consume larger quantities of alcohol compared to female drinkers and are more likely to experience behavioral problems related to their alcohol use [42]. These patterns suggest that alcohol use and its associated risks are not only influenced by gender but also by the cultural norms that define acceptable behaviors for men and women [42]. Understanding these gender and cultural dynamics is crucial for designing effective interventions to address alcohol-related issues, particularly among vulnerable populations.

### Limitation

There are some limitations associated with this study. Firstly, the study was health facility-based and only included PLHIV who had clinical appointments and showed up for their appointments during the study period. Potential participants who were initially sampled and didn’t show up for their appointment were excluded. Also, some sampled patients refused to participate in the study due to time limitations and other commitments. The respondents included in this study were limited to those who could speak the commonly spoken languages of English, Afrikaans, and Oshiwambo. There are many more languages spoken in Namibia, and patients were excluded as a result. Our data was also based on self-reporting, which has the potential to introduce social desirability bias. Also, since participants were required to recall past events or information, there is potential for information bias.

Another potential limitation of our analysis is the risk of multicollinearity, particularly due to the inclusion of depression, alcohol misuse, and suicidality as independent variables in separate logistic regression models. Since these mental health outcomes are often interrelated [43, 44]. Their inclusion could lead to inflated standard errors, making it challenging to isolate the individual effects of demographic and clinical predictors. However, we observed no such inflated standard errors, nor high variance inflation factors (VIF was <5% for this study, which indicates low multicollinearity), or unusually wide confidence intervals around the regression coefficients in our models. Nevertheless, the difficulty in pinpointing the exact causal pathways between these three interrelated variables remains a consideration when interpreting our findings.

### Conclusion [45]

Our study findings indicate that Namibia has a higher prevalence of suicidality and alcohol use disorder among PLHIV in comparison to other sub-Saharan countries, but the prevalence of depression is comparatively lower. The relatively lower prevalence of depression, in comparison to suicidality and alcohol use disorder (AUD) among PLHIV in Windhoek, may be explained by the fact that many participants had been on ART for five years or longer. Some studies suggest that the risk of depression is typically highest shortly after an HIV diagnosis [46, 47]. Furthermore, some studies reported lower depression rates in urban areas compared to rural settings [45]; however, further research is needed to better understand and confirm this trend, as other studies reported the opposite [48]. Having noted that, except for sex, there are no other risk factors that can be directly correlated with mental health illnesses amongst PLHIV, as findings vary across different populations of PLHIV and diverse contextual settings. Screening all PLHIV for mental illnesses, regardless of demographic characteristics, should be initiated early in the patients’ ART journey. The regular screening of PLHIV is essential for early diagnosis and timely treatment of mental illnesses. This proactive approach will help prevent mental health conditions from adversely affecting HIV care outcomes in this population.

## Supplementary Information

Below is the link to the electronic supplementary material.

Supplementary material 1 (XLSX 19.0 kb)

## Data Availability

Study data is available on request.

## Code Availability

Codes are available on request.

## References

[1] Kharsany AB, Karim QA. HIV infection and AIDS in sub-saharan Africa: current status, challenges and opportunities. The open AIDS journal. 2016;2016(10):34–48. 10.2174/1874613601610010034 PMC4893541 27347270

[2] Remien RH, Stirratt MJ, Nguyen N, Robbins RN, Pala AN, Mellins CA. Mental health and HIV/AIDS: the need for an integrated response. AIDS. 2019;33(9):1411. 30950883 10.1097/QAD.0000000000002227 PMC6635049

[3] Park-Lee E, Lipari RN, Hedden SL, Kroutil LA, Porter JD. CBHSQ Data Review. Rockville (MD): Substance Abuse and Mental Health Services Administration (US); 2012. pp. 1–35. Receipt of Services for Substance Use and Mental Health Issues Among Adults: Results from the 2016 National Survey on Drug Use and Health 29431966

[4] Gaynes BN, Pence BW, Eron JJ, Miller WC. Prevalence and comorbidity of psychiatric diagnoses based on reference standard in an HIV+ patient population. Psychosom Med. 2008;70(4):505. 18378865 10.1097/PSY.0b013e31816aa0cc PMC2900836

[5] Bhatia M, Munjal S. Prevalence of depression in people living with HIV/AIDS undergoing ART and factors associated with it. J Clin Diagn Research: JCDR. 2014;8(10):WC01. 10.7860/JCDR/2014/7725.4927 PMC4253251 25478433

[6] Niu L, Luo D, Liu Y, Silenzio VM, Xiao S. The mental health of people living with HIV in China, 1998–2014: a systematic review. PLoS One. 2016;11(4): e0153489. 27082749 10.1371/journal.pone.0153489 PMC4833336

[7] Kinyanda E, Hoskins S, Nakku J, Nawaz S, Patel V. Prevalence and risk factors of major depressive disorder in HIV/AIDS as seen in semi-urban Entebbe district, Uganda. BMC Psychiatry. 2011;11(1):1–9. 22208452 10.1186/1471-244X-11-205 PMC3260105

[8] Jonsson G, et al. Management of mental health disorders in HIV-positive patients. South Afr J HIV Med. 2013;14(4):155–65.

[9] (2018). *Namibia Population-Based HIV Impact Assessment (NAMPHIA)*.

[10] Sheehan D, et al. The mini international neuropsychiatric interview (MINI). A short diagnostic structured interview: reliability and validity according to the CIDI. Eur Psychiatry. 1997;12(5):224–31.

[11] Sheehan DV, et al. The Mini-International Neuropsychiatric Interview (M.I.N.I.): the development and validation of a structured diagnostic psychiatric interview for DSM-IV and ICD-10, (in eng). J Clin Psychiatry. 1998;59(20):22–3. 9881538

[12] Egbe CO, Dakum PS, Ekong E, Kohrt BA, Minto JG, Ticao CJ. Depression, suicidality, and alcohol use disorder among people living with HIV/AIDS in Nigeria. BMC Public Health. 2017;17(1):1–13. 28577548 10.1186/s12889-017-4467-5 PMC5457576

[13] Hartzler B, et al. Prevalence and predictors of substance use disorders among HIV care enrollees in the united States. AIDS Behav. 2017;21:1138–48. 27738780 10.1007/s10461-016-1584-6 PMC6089366

[14] Opoku Agyemang S, et al. Prevalence and associations of depression, anxiety, and stress among people living with HIV: a hospital-based analytical cross-sectional study. Health Sci Rep. 2022;5(5): e754. 10.1002/hsr2.754. 35949667 10.1002/hsr2.754 PMC9358537

[15] Chander G, Himelhoch S, Moore RD. Substance abuse and psychiatric disorders in HIV-positive patients. Drugs. 2006;66(6):769–89. 16706551 10.2165/00003495-200666060-00004

[16] Besa N, Paul R, Hachaambwa L. Psychiatric symptoms among an HIV positive urban population in Lusaka, Zambia. Med J Zambia. 2015;42(2):84–9.

[17] Whiteman K, Ruggiano N, Thomlison B. Transforming mental health services to address gender disparities in depression risk factors. J Women Aging. 2016;28(6):521–9. 10.1080/08952841.2015.1072027. 27391089 10.1080/08952841.2015.1072027 PMC5661879

[18] Burt VK, Stein K. Epidemiology of depression throughout the female life cycle. J Clin Psychiatry. 2002;63:9–15. 11995779

[19] Kessler RC, Berglund P, Demler O, Jin R, Merikangas KR, Walters EE. Lifetime prevalence and age-of-onset distributions of DSM-IV disorders in the National comorbidity survey replication. Arch Gen Psychiatry. 2005;62(6):593–602. 15939837 10.1001/archpsyc.62.6.593

[20] Cyranowski JM, Frank E, Young E, Shear MK. Adolescent onset of the gender difference in lifetime rates of major depression: a theoretical model. Arch Gen Psychiatry. 2000;57(1):21–7. 10632229 10.1001/archpsyc.57.1.21

[21] Bernard C, Dabis F, de Rekeneire N. Prevalence and factors associated with depression in people living with HIV in sub-Saharan africa: a systematic review and meta-analysis. PLoS One. 2017;12(8):e0181960. 28783739 10.1371/journal.pone.0181960 PMC5544236

[22] Bernard C, et al. Prevalence and factors associated with severe depressive symptoms in older west African people living with HIV, (in eng). BMC Psychiatry. 2020;20(1):442. 10.1186/s12888-020-02837-0. 32912173 10.1186/s12888-020-02837-0 PMC7481548

[23] Deshmukh NN, Borkar AM, Deshmukh JS. Depression and its associated factors among people living with HIV/AIDS: can it affect their quality of life? J Family Med Prim Care. 2017;6(3):549–53. 10.4103/2249-4863.222016. 29417006 10.4103/2249-4863.222016 PMC5787953

[24] Chan BT, et al. Prevalence and correlates of psychosocial conditions among people living with HIV in southern India. AIDS Care. 2017;29(6):746–50. 10.1080/09540121.2016.1231887. 27643850 10.1080/09540121.2016.1231887 PMC5552362

[25] Kuria MW, et al. The association between alcohol dependence and depression before and after treatment for alcohol dependence, (in eng). ISRN Psychiatry. 2012;2012:482802. 10.5402/2012/482802. 23738204 10.5402/2012/482802 PMC3658562

[26] Felker-Kantor EA, Wallace ME, Madkour AS, Duncan DT, Andrinopoulos K, Theall K, Stigma HIV. Mental health, and alcohol use disorders among people living with HIV/AIDS in new orleans, (in eng). J Urban Health. 2019;96(6):878–88. 10.1007/s11524-019-00390-0. 31520231 10.1007/s11524-019-00390-0 PMC6904691

[27] Tsai YT, et al. Suicidality Among People Living With HIV From 2010 to 2021: A Systematic Review and a Meta-regression, (in eng). Psychosom Med. 2022;84(8):924–39. 10.1097/psy.0000000000001127. 36162070 10.1097/PSY.0000000000001127 PMC9553271

[28] Pelton M, et al. Rates and risk factors for suicidal ideation, suicide attempts and suicide deaths in persons with HIV: a systematic review and meta-analysis. Gen psychiatry. 2021;34(2):1. 10.1136/gpsych-2020-100247 PMC8042999 33912798

[29] Hendershot CS, Stoner SA, Pantalone DW, Simoni JM. Alcohol use and antiretroviral adherence: review and meta-analysis. J Acquir Immune Defic Syndr. 2009;52(2):180–202. 10.1097/QAI.0b013e3181b18b6e. 19668086 10.1097/QAI.0b013e3181b18b6e PMC2815237

[30] Kalichman SC, Katner H, Hill M, Kalichman MO, Hernandez D. Alcohol-related intentional antiretroviral nonadherence among people living with HIV: test of an interactive toxicity beliefs process model. J Int Assoc Provid AIDS Care. 2019;18: 2325958219826612. 10.1177/2325958219826612. 30782051 10.1177/2325958219826612 PMC6748551

[31] Fatch R, Emenyonu NI, Muyindike W, Kekibiina A, Woolf-King S, Hahn JA. Alcohol interactive toxicity beliefs and ART non-adherence among HIV-infected current drinkers in Mbarara, Uganda. AIDS Behav. 2017;21:1812–24. 27198557 10.1007/s10461-016-1429-3 PMC5116271

[32] Kibera AW, Kuria MW, Kokonya DA. Alcohol Use Disorders Among HIV and AIDS Patients at Kenyatta National Hospital (KNH) Comprehensive Care Centre, Nairobi, Kenya, 2017.

[33] Rukundo GZ, Wakida EK, Karungi CK, Asasira J, Kumakech E, Obua C. Depression, suicidality, substance-use and associated factors among people living with HIV the COVID-19 pandemic in Uganda. PLoS One. 2023;18(5):e0285310. 37146057 10.1371/journal.pone.0285310 PMC10162553

[34] Necho M, Belete A, Getachew Y. The prevalence and factors associated with alcohol use disorder among people living with HIV/AIDS in Africa: a systematic review and meta-analysis, (in eng). Subst Abuse Treat Prev Policy. 2020;15(1):63. 10.1186/s13011-020-00301-6. 32831129 10.1186/s13011-020-00301-6 PMC7444054

[35] Necho M, Belete A, Getachew Y. The prevalence and factors associated with alcohol use disorder among people living with HIV/AIDS in Africa: a systematic review and meta-analysis. Subst Abuse Treat Prev Policy. 2020;15:1–15. 32831129 10.1186/s13011-020-00301-6 PMC7444054

[36] WanjaKibera A, Kuria MW, Kokonya DA. Alcohol Use Disorders Among HIV and AIDS Patients at Kenyatta National Hospital (KNH) Comprehensive Care Centre, Nairobi, Kenya, 2017.

[37] Duko B, Ayalew M, Ayano G. The prevalence of alcohol use disorders among people living with HIV/AIDS: a systematic review and meta-analysis. Subst Abuse Treat Prev Policy. 2019;14(1):52. 10.1186/s13011-019-0240-3. /11/14 2019. 31727086 10.1186/s13011-019-0240-3 PMC6854786

[38] Farley J, et al. Screening for hazardous alcohol use and depressive symptomatology among HIV-infected patients in Nigeria: prevalence, predictors, and association with adherence. J Int Assoc Physicians AIDS Care (Chic). 2010;9(4):218–26. 10.1177/1545109710371133. 20798401 10.1177/1545109710371133 PMC2951272

[39] Goar SG, Audu MD, Agbir MT, Dochalson E. Prevalence and socio-demographic correlates of alcohol use disorders among HIV patients. Afr J Drug Alcohol Stud. 2011;10(1):1.23024611

[40] Morojele NK, Kekwaletswe CT, Nkosi S. Associations between alcohol use, other psychosocial factors, structural factors and antiretroviral therapy (ART) adherence among South African ART recipients. AIDS Behav. 2014;18:519–24. 23934270 10.1007/s10461-013-0583-0

[41] Bravo F, Gual A, Lligoña A, Colom J. Gender differences in the long-term outcome of alcohol dependence treatments: an analysis of twenty‐year prospective follow up. Drug Alcohol Rev. 2013;32(4):381–8. 23240781 10.1111/dar.12023

[42] Grant BF, et al. Epidemiology of DSM-5 alcohol use disorder: results from the National epidemiologic survey on alcohol and related conditions III. JAMA Psychiatr. 2015;72(8):757–66. 10.1001/jamapsychiatry.2015.0584 PMC5240584 26039070

[43] Berglund M, Ojehagen A. The influence of alcohol drinking and alcohol use disorders on psychiatric disorders and suicidal behavior. Alcoholism: Clin Experimental Res. 1998;22:s333–45. 10.1097/00000374-199807001-00010 9799958

[44] Coêlho BM, Andrade LH, Guarniero FB, Wang Y-P. The influence of the comorbidity between depression and alcohol use disorder on suicidal behaviors in the São Paulo epidemiologic catchment area study, Brazil. Rev Bras Psiquiatr. 2010;32(4):396–408. 21308261 10.1590/s1516-44462010005000027

[45] Saha A, Mandal B, Muhammad T, Ali W. Decomposing the rural–urban differences in depression among multimorbid older patients in India: evidence from a cross-sectional study. BMC Psychiatry. 2024;24(1): 60. 10.1186/s12888-023-05480-7. 38254089 10.1186/s12888-023-05480-7 PMC10804604

[46] Madundo K, Knettel BA, Knippler E, Mbwambo J. Prevalence, severity, and associated factors of depression in newly diagnosed people living with HIV in Kilimanjaro, Tanzania: a cross-sectional study. BMC Psychiatry. 2023;23(1): 83. 10.1186/s12888-022-04496-9. 36726113 10.1186/s12888-022-04496-9 PMC9890688

[47] Liu Z, Chen X, Li J, Xie Z, Huang Y, Luo D. HIV-related stress predicts depression over five years among people living with HIV. Front Public Health. 2023;11:1163604. 37377546 10.3389/fpubh.2023.1163604 PMC10291293

[48] Breslau J, Marshall GN, Pincus HA, Brown RA. Are mental disorders more common in urban than rural areas of the United States?? J Psychiatr Res. 2014;56:50–5. 10.1016/j.jpsychires.2014.05.004. 24857610 10.1016/j.jpsychires.2014.05.004

